# Soluble Salts in Processed Cheese Prepared with Citrate- and Phosphate-Based Calcium Sequestering Salts

**DOI:** 10.3390/molecules29153631

**Published:** 2024-07-31

**Authors:** Gaurav Kr Deshwal, Laura G. Gómez-Mascaraque, Mark Fenelon, Thom Huppertz

**Affiliations:** 1 Department of Food Chemistry and Technology, Teagasc Food Research Centre, P61 C996 Fermoy, Ireland; 2 Department of Agrotechnology and Food Sciences, Wageningen University, Bornse Weilanden 9, 6708 WG Wageningen, The Netherlands; 3 Department of Food and Nutritional Sciences, University College Cork, T12 YN60 Cork, Ireland

**Keywords:** water-soluble extract, 10 kDa-permeable Ca, non-sedimentable protein

## Abstract

In this study, the protein and salts distribution (Ca, P, Na and Mg) in processed cheese (PC) samples prepared with 180 or 360 mEq/kg of the calcium sequestering salts (CSS) disodium phosphate (DSP), disodium pyrophosphate (DSPP), sodium hexametaphosphate (SHMP) and trisodium citrate (TSC) was studied. For this purpose, a water-soluble extract (WSE) of PC samples was prepared. All PC samples contained 45–46% moisture, 26–27% fat and 20–21% protein and had a pH of 5.2 or 5.7. Ultracentrifugation slightly reduced the protein content of the WSE of PC, indicating that most protein in the WSE was non-sedimentable. At equal concentration of CSS, the protein content of the WSE was higher for PC at pH 5.7 compared to PC at pH 5.2. Approximately 55–85% of the Ca and P in the WSE of samples was 10 kDa-permeable for PC prepared with DSPP and SHMP. This suggests that the formation of non-permeable Ca–polyphosphate–casein complexes. For PC prepared with TSC, >90% of Ca in the WSE was 10 kDa-permeable, indicating that micellar disruption arises from sequestration of micellar Ca. These results indicate that the WSE method is an appropriate method to understand how salts present in PC are distributed. However, the WSE and ultracentrifugal supernatant of the WSE can include both soluble and protein-associated salts. Therefore, determining levels of salts in 10 kDa permeate of ultracentrifugal supernatant of the WSE is most appropriate.

## 1. Introduction

Processed cheese (PC) is a viscoelastic matrix obtained by mixing and heating natural cheese(s), calcium sequestering salts (CSS) and additional dairy ingredients (e.g., butter, anhydrous milk fat and milk powders), non-dairy ingredients and additives (acidulants, stabilizers, colors and flavors) [1]. The CSS typically used in processed cheese manufacture consist of monovalent cations (H^+^, Na^+^ or K^+^) and polyvalent anions (citrate or phosphates). As a result of Ca sequestration, some Ca in the casein micelles is exchanged with the monovalent cations of the CSS [2,3]. Citrates and phosphates are the most commonly used CSS. They decrease the concentration of free calcium ions by sequestering Ca. The Ca-binding ability of CSS follows the order long-chain phosphates > tri-polyphosphates > pyrophosphates > citrates > orthophosphates [2,3]. Furthermore, CSS can also dissociate the casein micelles, thereby resulting in increased hydration and voluminosity of the casein micelles [4].

Whereas the distribution of salts between the soluble and insoluble phase of liquid dairy products, such as milk, is readily determined using fractionation methods based on centrifugation and filtration techniques [5], determining salt distributions in cheese and cheese products is far more challenging [6,7]. However, being able to do so is important, because insoluble Ca, i.e., the amount of Ca within the casein matrix in PC, has been reported to play an important role in modulating the textural and functional properties of PC [8]. The concentration of soluble salts in cheese matrices has been estimated previously by hydraulically pressing the cheese sample under up to 6 tons of pressure and measuring the salts in the obtained liquid, which is generally referred as cheese juice [9]. This cheese juice method has been used to study the effect of proteolysis on salt equilibria in cheddar cheese and the amount of obtained cheese juice was found to decrease with age of cheese [8]. Water-soluble extraction is an alternative method has been used to quantify the levels of soluble salts in cheese, including in PC [9]. Hassan et al. [9] compared the cheese juice method and the water-soluble extraction method and reported similar levels of soluble Ca for the juice method and the extraction method when mixing of cheese and water in a 1:1.5 ratio.

Several studies have evaluated the effect of CSS on the solubilization of caseins and salts in rennet casein [10] and micellar casein isolate (MCI) [11] using centrifugation (30,000–100,000× *g*) of samples. For MCI, it was shown that the Ca in the ultracentrifugal supernatant consisted of both soluble and protein-associated Ca [11]. In the case of PC, researchers have measured the Ca and P level in water-soluble extract (WSE) of PC prepared with different mixtures of CSS [2,3]. However, in line with aforementioned findings for MCI [11], the WSE of PC may also include both soluble and protein-associated salts. Hence, the Ca levels of the WSE alone do not provide conclusive information on the level of soluble Ca with reference to the PC matrix. Further information may be obtained by filtration of the WSE of PC through a membrane having a pore size <10 kDa [12]. The analysis of supernatants and 10 kDa permeates of micellar casein isolate (MCI) added with different CSS allowed for the quantification of protein-bound sedimentable, protein-bound non-sedimentable and 10 kDa-permeable Ca. This approach helped in gaining insights about the mode of action of CSS and formation of complexes between Ca and CSS [11].

Therefore, to fully understand the state of salts in the PC matrix, it is also important to consider the non-sedimentable fractions and 10 kDa-permeable fractions. This was undertaken in the current study. The aim of the present study was to understand the distribution of salts in a PC matrix (45% moisture, 26% fat and 21% protein) prepared with 180 or 360 mEq/kg disodium phosphate (DSP), disodium pyrophosphate (DSPP), sodium hexametaphosphate (SHMP) or trisodium citrate (TSC) at pH 5.2 and 5.7. Outcomes hereof aid the understanding the mode of action of CSS in a PC matrix.

## 2. Results

### 2.1. Cheese Composition and pH

The Gouda cheese contained 41.8% moisture, 30.2% fat and 22.3% protein, whereas all PC samples contained 45–46% moisture, 26–27% fat and 20–21% protein (Table 1), with no significant differences observed between the different PC samples (*p* > 0.05). Furthermore, Ca (171–177 mM) and Mg (9.7–10.7 mM) concentrations also did not differ significantly between PC samples (*p* > 0.05). The concentration of P and Na varied significantly (*p* > 0.05) among PC samples (Table 1). This can be attributed to the different Na and P contents of the added CSS as well as the differences in the levels at which they were added.

### 2.2. Protein Partitioning

The amount of WSE obtained and the protein content of the WSE and the ultracentrifugal supernatant thereof are presented in Table 2. From the mixture of 15 g cheese and 30 g water used for obtaining the WSE, between 6 and 29 g of WSE was obtained (Table 2). For PC samples containing 180 mEq/kg of CSS, a significantly (*p* > 0.05) higher amount of WSE was obtained from PC samples at pH 5.2 than for PC samples at pH 5.7. For PC samples at pH 5.7, a higher concentration of CSS resulted in a significantly (*p* < 0.0.5) higher amount of WSE (Table 2).

The protein content of the WSE of all the PC samples, except sample PC-DSP-180-pH5.2, was higher than that of the WSE of Gouda cheese (Table 2). Increasing the concentration of CSS from 180 to 360 mEq/kg significantly (*p* < 0.05) increased the protein content of the WSE. At equal concentration of CSS, the protein content of the WSE was higher for PC at pH 5.7 compared to pH 5.2. The WSE of PC prepared with SHMP and TSC had significantly (*p* < 0.05) higher protein content than the WSE of PC prepared with DSP and DSPP (Table 2). The protein content of the ultracentrifugal supernatant of the WSE was slightly lower than that of the WSE itself (Table 2). This could be attributed to some sedimentation or some loss of protein with fat on ultracentrifugation.

### 2.3. Salts Partitioning

Table 3 presents the concentrations of Ca, P, Mg and Na in the WSE, the ultracentrifugal supernatant of the WSE and 10 kDa permeate of ultracentrifugal supernatant of the WSE of Gouda cheese and PC prepared therefrom with different CSS at pH 5.2 and 5.7. The concentrations of Ca and Mg were significantly (*p* < 0.05) higher in the WSE of Gouda cheese than in the WSE of the PC samples (Table 3). The concentration of Ca in the WSE of PC prepared with DSP decreased with increasing concentration of DSP, which is in line with previous results [4]. On increasing the concentration of SHMP in PC from 180 mEq/kg to 360 mEq/kg, the Ca concentration in the WSE increased significantly (*p* < 0.05) by almost 2-fold at both pH 5.2 and pH 5.7.

The concentrations of salts in the ultracentrifugal supernatant of the WSE did not differ significantly (*p* > 0.05) from those in the WSE (Table 3), indicating that there were only low levels of sedimentable salts in the WSE. The levels of Ca and P in the 10 kDa permeate of the WSE showed non-significant (*p* > 0.05) differences compared with their respective levels in the WSE for PC prepared with DSP and TSC. However, for PC prepared with DSPP and SHMP, levels of Ca and P in the 10 kDa-permeable fraction of the WSE were significantly lower (*p* < 0.05) than those in the WSE itself (Table 3). This difference in Ca content between the WSE and the 10 kDa fraction thereof was larger for WSE from PC samples at pH 5.7 than for WSE for PC samples at pH 5.2 (Table 3).

**Figure 1:**
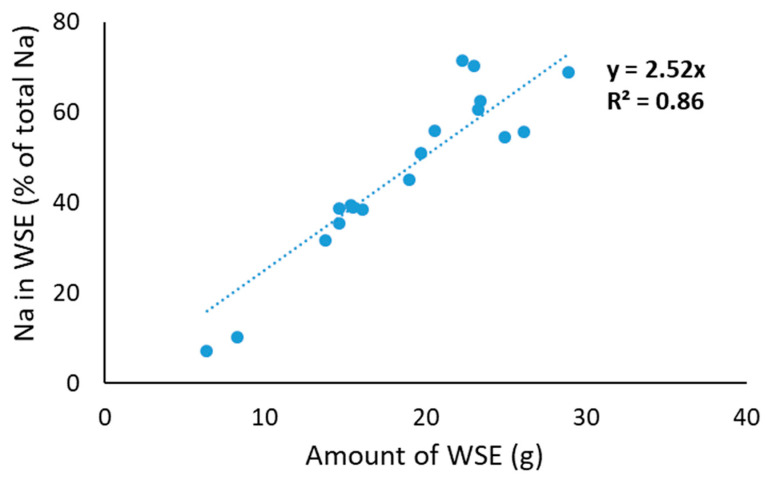
Correlation between amount of water-soluble extract (WSE) and amount of sodium in WSE.

In addition to concentrations of the different salts in the WSE, it is also important to understand what percentage of the total salts in PC is considered soluble. For this, we assumed that the concentration of salts in the water phase of the WSE is the same as the water that remains present in the residue from WSE preparation. To assess this, we used Na as a marker because it will not form specific complexes. When plotting the amount of WSE vs. the amount of sodium in the obtained WSE, indeed, a strong linear correlation was obtained, with a slope of 2.52 (Figure 1). This indicates that 100% of Na would be in the obtained WSE if ~39.7 g of WSE was obtained. This amount of 39.7 g WSE is in line with the expected amount of maximum WSE considering ~7 g of water from the 15 g of PC plus 30 g of water added, and some additional dry matter in the WSE in the form of protein (Table 2) and salts (Table 3). Hence, the % soluble for the other salts could be calculated as follows:

(1) $$\%\;soluble\;salts=\frac{Original\;\%salt\;in\;WSE}{Amount\;of\;WSE}\times Calculated\;WSE\;amount\;for\;100\%\;Na\;in\;WSE$$

The % soluble Ca (expressed as % of total Ca in cheese) of PC prepared with SHMP increased significantly (*p* < 0.05) with increasing concentration of SHMP at both pH 5.2 and pH 5.7 (Table 4). However, the percentage of Ca in the 10 kDa permeate (expressed as % of total Ca in the WSE) decreased significantly (*p* < 0.05) with increasing concentration of SHMP (Table 4), highlighting the formation of insoluble casein–Ca–hexametaphosphate complexes. Similarly, for PC prepared with DSP, the percentage of salts in the WSE (% of total in cheese) decreased significantly (*p* < 0.05) with increasing concentration of DSP (Table 4). This is in line with our previous studies on rennet casein [10] and micellar casein isolate [11]. On the other hand, the % of soluble Ca in 10 kDa permeate of PC prepared with TSC increased with increasing concentration at both pH 5.2 and pH 5.7 (Table 4). This indicates the formation of soluble Ca–citrate complexes. For PC prepared with TSC, ~90% of Ca in the WSE of was present in 10 kDa permeate (Table 4). This indicates that Ca in the WSE of processed cheese made with TSC is present in Ca–citrate complexes without casein. PC prepared with SHMP and DSPP had 54–94% of soluble Ca in the 10 kDa permeate, indicating the formation of insoluble Ca–pyro/polyphosphate complexes with casein (Table 4). The percentage of Ca in 10 kDa permeate, expressed as % of total Ca in WSE, was significantly (*p* < 0.05) lower at higher concentration of all studied CSS. This could be due to formation of a greater number of insoluble complexes among casein, Ca and CSS.

For PC prepared with 180 mEq/kg of DSP, DSPP, SHMP and TSC, a higher percentage of soluble Ca, P and Mg (expressed as a % of total Ca, P and Mg in PC) was found in PC at pH 5.2 as compared to at pH 5.7 (Table 4). For PC prepared with 360 mEq/kg of CSS, higher percentages of soluble Ca, P and Mg were obtained at pH 5.7 than at pH 5.2. With an increase in pH from 5.2 to 5.7, PC prepared with 360 mEq/kg DSPP or SHMP showed notably a higher increase in percentage of salts in WSE as compared to DSP or TSC. Higher percentage of Ca, P and Mg were present in 10 kDa permeate of all the PC at pH 5.2 as compared to pH 5.7 (Table 4).

## 3. Discussion

The methods to study the distribution of salts in cheese are fractionation, the WSE method, the cheese juice method and the titration method. The cheese juice method involves the use of pressure to extract the aqueous phase of cheese [9]. This method has been used to measure the changes during ripening, by evaluating the concentration of nitrogen, salts, lactose and lactic acid in the cheese juice [6]. The amount of juice decreases with increasing ripening time of natural cheese and can reach a point where no juice can be extracted from the cheese. This method has been described as the ideal method to study the mineral equilibrium in cheese as there is no dilution or solubilization of cheese components [9,13]. Based on our preliminary trials (unpublished), however, it was very difficult to obtain juice for some PC samples due to their smooth texture, which leads to PC samples passing through the pores of the base plate in a hydraulic press. An alternative is a fractionation method that involves centrifugation at 100,000 to 400,000× *g* to obtain the expressible serum for evaluating the soluble Ca in cheese [6]. However, centrifugal methods are only suitable for high-moisture cheese, like mozzarella [7]. For cheddar cheese, no serum was obtained even after ultracentrifugation at 100,000× *g* for 2 d [6]. The WSE method is based on dilution of cheese with water in ratios ranging from 1:1 to 1:10, mixing using a stomacher or ultra-turrax, followed by centrifuging the cheese–water mixture, filtering the supernatant, and measuring the concentration of salts in the filtrate [7]. Some of the potential problems associated with the WSE method are the changes in mineral equilibrium due to changes in pH, ionic strength and different ratios of cheese and water [9]. Higher dilution of cheese with water influenced the pH of the mix and hence the Ca equilibrium. There are possibilities of solubilizing Ca lactate crystals as a result of the pH shift caused by dilution [9]. The titration method is based on acid–base buffering curves obtained by plotting the buffering index as a function of pH [14]. Cheese samples are titrated from their initial pH to pH 3 with 0.5 M HCl and then back titrated to pH 9 with 0.5 M NaOH at 25 °C. During acidification of cheese, there is a buffering maximum at pH ~5 due to solubilization of colloidal calcium phosphate (CCP) and the area of this peak indicates the amount of CCP. The difference in peak areas of acid and base titration is used to calculate the amount of insoluble Ca [9]. An advantage of titration method is the presence of unique buffering peak for CCP, which is not affected by the formation of Ca lactate crystals. However, this method does not allow for differentiation between salts and other buffering compounds during acid–base titration [9,14]. For determining salts distributions in PC samples, we considered the WSE method most appropriate. However, also for the WSE method, it is important to understand how the salts present are associated.

Because the WSE and ultracentrifugal supernatant of the WSE can include both free and protein-associated salts, determining levels of salts in 10 kDa permeate of the WSE is required. Ultrafiltration membranes allow for the permeation of soluble salts while retaining proteins and associated salts [15]. Proteins present in the WSE were found to be in a form too small to sediment, because all the protein in the WSE was non-sedimentable (Table 2). However, not all the Ca and P in the WSE permeated through the 10 kDa membrane, suggesting the complexation of Ca with (non-sedimentable) casein. Deshwal et al. [11] previously showed that when TSC was added to 5% MCI, the level of 10 kDa-permeable Ca increased significantly. In contrast, the addition of phosphate-based CSS did not increase the 10 kDa-permeable Ca in MCI [11]. Our experiments confirmed these findings in the PC matrix (Table 3 and Table 4) and show that addition of 180 or 360 mEq/kg of SHMP caused a large increase in non-sedimentable Ca, but only a small increase in 10 kDa-permeable Ca. For PC prepared with 360 mEq/kg SHMP, only ~55% of Ca in the WSE was 10 kDa-permeable (Table 4). This highlights the formation of Ca–HMP–casein complexes. For the WSE from PC made with 360 mEq/kg TSC, Ca in 10 kDa permeate represents >90% of Ca in WSE (Table 4), indicating sequestration of micellar Ca. It is interesting to note the higher percentage of Ca in 10 kDa permeate of WSE of PC prepared with DSP or DSPP, which indicate possibilities of Ca–phosphate complexes being small enough to permeate a 10 kDa membrane (Table 3 and Table 4). This effect was more dominant at pH 5.2 than at pH 5.7, which could be due to additional acid-induced solubilization of micellar Ca at pH 5.2.

The lowest amount of WSE for PC prepared with SHMP or TSC (Table 2) indicates that a greater amount of water was entrapped in their matrices, whereas these samples showed the highest amount of protein and salts in the WSE (Table 3). Mizuno and Lucey [16] reported that more intensive casein dispersion increases the protein hydration and fat emulsification, resulting in higher intensity of casein cross-links. Higher emulsification of fat reduces the fat globule size and enhances their incorporation in the protein matrix. These protein-coated fat globules act like large protein units and assist in formation of PC matrix and are transferred to the pellet fraction of the WSE after ultracentrifugation [17]. SHMP has been reported to be more effective in promoting fat emulsification even at a low degree of casein dispersion, leading to higher ability of casein to re-associate and form cross-links, thereby reducing melting ability of PC [3]. The abovementioned process can explain the reduction in protein content of ultracentrifugal supernatant of the WSE of PC prepared with SHMP and TSC. The amount and protein content of WSE was strongly dependent on pH, with higher protein content in the WSE at higher pH for a given type and concentration of CSS (Table 2). With increasing pH, the net-negative charge of protein increases resulting in more repulsion among the proteins increased casein hydration and caused the formation of a more open structure, and subsequently a higher amount of protein in the WSE [3,18]. A study reported that PC made with DSP showed the lowest casein solubilization and highest hardness as compared to PC prepared with SHMP and TSC [4]. Moreover, hardness of PC prepared with DSP and TSC has been reported to correlate highly with protein content of WSE and soluble Ca [19].

The addition of CSS to natural cheese results in the dispersion of caseins induced by a loss of Ca phosphate cross-links and the formation of different types of soluble or insoluble Ca–CSS complexes with or without caseins [16]. This disruption of casein micelles is highlighted by increased protein content of the WSE of PC with added CSS (Table 2). The Ca sequestration of CSS follows the following order: SHMP > DSPP > TSC > DSP [10,11], which is consistent with the protein content of the WSE in the present study. In our previous studies on rennet casein and MCI, we reported that DSP forms insoluble Ca–phosphate complexes (e.g., Ca_3_(PO_4_)_2_ or CaHPO_4_). SHMP complexes with Ca and casein simultaneously, forming insoluble casein–Ca–polyphosphate complexes. SHMP can form complexes directly with casein, even in absence of Ca. TSC forms soluble Ca–citrate complexes and solubilizes phosphate between pH 5.5 and 6.5 [10,11]. Similar findings were apparent from the results in the present study in a PC matrix. CSS can disrupt casein micelles in three different ways: binding the micellar Ca, peptization of nanoclusters and disruption of protein–protein interactions [15]. Solubilization of micellar Ca phosphate can be measured from the 10 kDa-permeable fraction instead of the non-sedimentable fraction or WSE, because WSE includes both soluble and protein-associated salts [20]. The loss of Ca cross-links due to the action of CSS in natural cheese increases the protein content of the WSE, reduces the protein–protein interactions and subsequently reduces the hardness and increases the tendency to melt. However, textural and rheological attributes of PC are not only influenced by casein and Ca solubilization but also depend on the mechanism of action of CSS [19].

## 4. Materials and Methods

### 4.1. Materials

Lactic acid (80% in aqueous solution) was purchased from VWR International (Dublin, Ireland). Sodium hydroxide (8 N), DSP (CAS-No. 7558-79-4), DSPP (CAS-No. 7758-16-9), SHMP (CAS No. 10124-56-8) and TSC (CAS-No. 68-04-2) were procured from Sigma-Aldrich (St. Louis, MO, USA). Nitric acid (CAS-No. 7697-37-2) of ppb-trace analysis grade (>69% purity) was supplied by OCON Chemicals Ltd. (Cork, Ireland).

### 4.2. Manufacture of Processed Cheese

A vacuum-packed block of rindless Gouda cheese (moisture 42.0%, protein 22.3%, fat 30.2% and salt 1.6%) was supplied by FrieslandCampina (Amersfoort, The Netherlands) and stored at 4 °C. All the PC samples were prepared from the same block of Gouda cheese. PC was prepared using a 2 L capacity Thermomix^®^ Vorwerk TM6-1, Wuppertal, Germany as per the methodology [4]. The temperature of heating vessel of the Thermomix was set at 90 °C, which took around 1 min to reach. The PC samples were prepared at 90 °C since it indicates the processing conditions used for manufacturing PC. The calculated amount of CSS and water (Table 5) for a final PC weight of 300 g was mixed using a magnetic stirrer at room temperature for 60 min, prior to its addition to the cheese. The calculated amount of comminuted Gouda cheese (Table 5) was mixed with continuous addition of the CSS and water mixture for 1 min at speed 2 (200 rpm). Water was added to achieve 45% moisture in the final PC. The amount of acid/base required to adjust the final pH was also considered in the calculated water required for moisture adjustment. The speed was increased to 3 (500 rpm) for 1 min and speed 5 (2000 rpm) for 1 min and 15 s. The surface of the heating vessel was scraped with a ladle to remove cheese particles from its sidewalls. The product was mixed for 15 s at speed 3 (500 rpm) and then at speed 5 (rpm 2000) for 30 s. The heating vessel was set at speed 3 (500 rpm) and 80 °C prior to pH adjustment. The calculated quantity of 80% lactic acid or 8 N NaOH required to reach pH 5.7 and pH 5.2 of PC was added dropwise with continuous mixing as the temperature of the heating vessel reached 80 °C. The pH values of PC manufactured in this study are typical values of PC manufactured at industrial scale. The speed was increased to 5 (rpm 2000) for 1 min for proper mixing of the added lactic acid or NaOH. PC samples were poured in plastic screw-cap containers (capacity 150 mL) and immediately transferred to a refrigerator at 5 °C.

The acronyms and numbers in sample codes (Table 5) represent the CSS used for manufacturing PC and its concentration, respectively. The number after the letters “pH” represents the pH of the PC sample. For example, PC-DSP-180-pH5.2 represents PC manufactured with 180 mEq/kg of DSP having pH 5.2.

### 4.3. Preparation of the Water-Soluble Extract and Fractions Thereof

The WSE of samples was obtained as per the methodology in [4]. Cheese samples and water (45–50 °C) were mixed at a 1:2 ratio and blended using a stomacher for 5 min. The obtained homogenate was kept in a water bath at 45 °C for 60 min, and subsequently centrifuged at 3000× *g* for 30 min at 4 °C and filtered through glass wool. The obtained filtrate was termed WSE.

**Figure 2:**
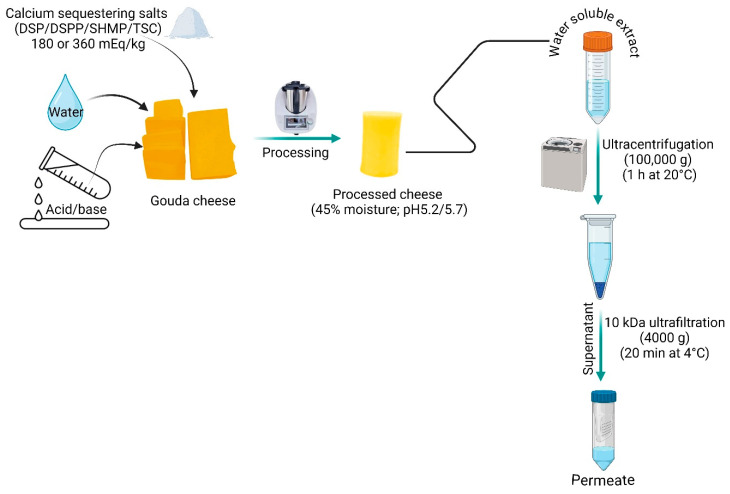
Experimental set-up for evaluating the salt speciation in processed cheese.

The non-sedimentable and sedimentable fractions of the WSE were separated using ultracentrifugation at 100,000× *g* for 1 h at 20 °C. The sedimentable (pellet) and non-sedimentable (supernatant) fraction were separated by decanting and weighed. A portion of supernatant was transferred to an Amicon^®^ Ultra15 centrifugal filter tube with a 10 kDa molecular mass cut-off membrane (Merck KGaA, Darmstadt, Germany) and centrifuged at 4000× *g* for 20 min at 20 °C. The obtained permeate was called the 10 kDa permeate and was used for salts analysis (Figure 2).

### 4.4. Analytical Methods

#### 4.4.1. Moisture, Protein and pH

The moisture content of samples was measured using a rapid microwave moisture analyzer (Model-Turbo Smart 5, CEM Corporation, Charlotte, NC, USA). The protein content of cheese and WSE was measured using the Kjeldahl method [21]. The pH of the cheese samples was measured after mixing 20 g comminuted sample with 12 g deionized water at 45 °C for 1 min using a stomacher (VWR Star-Blender LB400, VWR International BVBA, Leuven, Belgium).

#### 4.4.2. Salts Analysis

Inductively coupled plasma–optical emission spectrometry (ICP-OES) was used to determine the levels of Ca, P, Mg and Na in cheese, WSE, supernatant and 10 kDa permeate as per the methodology in [22]. About 2 g of cheese, WSE, supernatant or 10 kDa permeate was accurately weighed in Teflon cylindrical tubes and a volume of 5 mL concentrated HNO_3_ was added. Samples were digested at 180 °C (1600 W) for 15 min using a microwave digester. The digested samples were allowed to cool down to room temperature and volume was made to 100 mL using deionized water. The ICP-OES analysis was performed using an Agilent 5110 synchronous vertical dual-view ICP-OES analyzer (Agilent Technologies, Santa Clara, CA, USA). Prior to measurement, the instrument was calibrated with element standards (ICP standards prepared in 2–5% HNO_3_ matrix, REICCAL 10CR5, Reagecon, Shannon, Ireland) by setting correlation coefficient limit at ≥0.999. Yttrium (Y) and cesium (Cs) solution (0.4 mL Y and 10 mL Cs made up to 100 mL using 5% HNO_3_) were used as an internal standard and ionization buffer, respectively.

### 4.5. Statistical Analysis

All the experiments were performed in triplicate and the average and standard deviations are reported. Statistical difference of the mean values of the samples was determined at 5% level of significance using SPSS software (version 29, IBM, Armonk, NY, USA). One-way ANOVA was performed to obtain the mean squares and *p*-values for the effect of experimental factors (pH of PC and type and concentration of CSS) on the amount of soluble salts. Results were considered significant at *p* < 0.05. Duncan’s multiple range test was used to separate among the means of three replicates.

## 5. Conclusions

This study demonstrated the importance of mineral measurement of WSE, supernatant and 10 kDa permeate of PC to fully understand the interactions of CSS and casein micelles in a PC matrix. The highest amount of protein in WSE of PC prepared with SHMP signifies the maximum disintegration of casein micelles. Based on the percentage of 10 kDa-permeable Ca, expressed as percentage of Ca in WSE, TSC solubilized the micellar Ca. The formation of Ca–CSS complexes with or without casein was dependent on the CSS type and pH of PC. Higher levels of salts were present in 10 kDa permeate of all the PC at pH 5.2 as compared to pH 5.7. Future research should be aimed at comparing different methods of extracting and measuring the water-soluble fractions in a PC matrix. The knowledge gained will elucidate the key role of CSS in modulating casein hydration and dispersion during the manufacture of PC. Moreover, the findings may help in understanding the casein–CSS interactions and their subsequent influence on the textural and rheological attributes of PC.

## Figures and Tables

**Table 1:** Moisture, fat and protein content (all in %, *w*/*w*) and Ca, P, Mg and Na content (all in mM) of Gouda cheese and processed cheese (PC) prepared therefrom with 180 or 360 mEq/kg of the calcium sequestering salts (CSS) disodium phosphate (DSP), disodium pyrophosphate (DSPP), sodium hexametaphosphate (SHMP) or trisodium citrate (TSC) and adjusted to pH 5.2 or 5.7. Values are mean ± standard deviation (*n* = 3).

Sample	Moisture (%)	Fat (%)	Protein (%)	Ca (mM)	P (mM)	Mg (mM)	Na (mM)
Gouda cheese	41.82 ± 0.15 ^a^	30.24 ± 0.12 ^b^	22.27 ± 0.18 ^b^	194.5 ± 0.7 ^c^	155.5 ± 1.5 ^c^	11.56 ± 0.1 ^b^	231.8 ± 2.2 ^a^
PC-DSP-180-pH5.2	45.79 ± 0.32 ^b^	26.60 ± 0.15 ^a^	20.77 ± 0.04 ^a^	177.6 ± 0.1 ^b^	203.3 ± 0.3 ^e^	10.6 ± 0.1 ^a^	381.1 ± 1.1 ^b^
PC-DSPP-180-pH5.2	45.63 ± 0.34 ^b^	27.45 ± 0.06 ^a^	20.58 ± 0.06 ^a^	177.2 ± 0.1 ^b^	229.4 ± 0.1 ^f^	10.3 ± 0.1 ^a^	390.7 ± 0.5 ^bc^
PC-SHMP-180-pH5.2	45.90 ± 0.15 ^b^	26.53 ± 0.02 ^a^	20.87 ± 0.04 ^a^	173.2 ± 0.7 ^a^	317.8 ± 0.4 ^j^	10.2 ± 0.1 ^a^	472.1 ± 1.0 ^g^
PC-TSC-180-pH5.2	45.81 ± 0.20 ^b^	26.27 ± 0.12 ^a^	20.69 ± 0.05 ^a^	172.0 ± 0.9 ^a^	144.4 ± 0.2 ^b^	10.3 ± 0.1 ^a^	437.3 ± 7.8 ^e^
PC-DSP-360-pH5.2	45.89 ± 0.19 ^b^	26.28 ± 0.24 ^a^	20.72 ± 0.07 ^a^	174.7 ± 0.4 ^a^	260.3 ± 0.4 ^h^	10.5 ± 0.1 ^a^	523.1 ± 0.6 ^h^
PC-DSPP-360-pH5.2	46.08 ± 0.09 ^b^	26.50 ± 0.46 ^a^	20.72 ± 0.16 ^a^	172.8 ± 1.5 ^a^	310.7 ± 0.7 ^i^	10.1 ± 0.1 ^a^	473.4 ± 1.5 ^g^
PC-SHMP-360-pH5.2	45.92 ± 0.20 ^b^	25.73 ± 0.01 ^a^	20.79 ± 0.07 ^a^	170.5 ± 1.8 ^a^	497.9 ± 2.4 ^m^	10.2 ± 0.2 ^a^	633.5 ± 6.4 ^l^
PC-TSC-360-pH5.2	45.65 ± 0.30 ^b^	25.81 ± 0.09 ^a^	20.70 ± 0.14 ^a^	171.8 ± 1.5 ^a^	137.3 ± 1.3 ^a^	10.2 ± 0.1 ^a^	560.5 ± 4.7 ^j^
PC-DSP-180-pH5.7	45.94 ± 0.63 ^b^	27.02 ± 0.11 ^a^	20.67 ± 0.04 ^a^	178.2 ± 0.9 ^b^	200.4 ± 0.5 ^d^	10.4 ± 0.1 ^a^	409.3 ± 2.1 ^d^
PC-DSPP-180-pH5.7	46.08 ± 0.13 ^b^	26.20 ± 0.22 ^a^	20.87 ± 0.06 ^a^	177.7 ± 0.3 ^b^	232.9 ± 0.9 ^g^	10.7 ± 0.1 ^a^	391.6 ± 1.7 ^c^
PC-SHMP-180-pH5.7	45.75 ± 0.33 ^b^	26.17 ± 0.20 ^a^	20.68 ± 0.10 ^a^	175.5 ± 0.1 ^ab^	321.8 ± 0.5 ^k^	10.3 ± 0.1 ^a^	458.2 ± 1.4 ^f^
PC-TSC-180-pH5.7	45.79 ± 0.32 ^b^	26.77 ± 0.05 ^a^	20.57 ± 0.05 ^a^	175.3 ± 0.2 ^ab^	145.2 ± 0.2 ^b^	10.2 ± 0.1 ^a^	454.1 ± 3.7 ^f^
PC-DSP-360-pH5.7	45.84 ± 0.18 ^b^	25.97 ± 0.11 ^a^	20.89 ± 0.05 ^a^	176.7 ± 0.3 ^b^	261.4 ± 0.2 ^h^	10.2 ± 0.1 ^a^	549.7 ± 1.9 ^i^
PC-DSPP-360-pH5.7	45.81 ± 0.17 ^b^	26.90 ± 0.25 ^a^	20.79 ± 0.02 ^a^	173.1 ± 1.3 ^a^	318.4 ± 1.0 ^j^	10.3 ± 0.1 ^a^	465.5 ± 6.7 ^f^
PC-SHMP-360-pH5.7	46.00 ± 0.02 ^b^	25.63 ± 0.18 ^a^	20.68 ± 0.04 ^a^	171.8 ± 0.2 ^a^	477.3 ± 0.4 ^l^	9.7 ± 0.1 ^a^	625.1 ± 6.7 ^l^
PC-TSC-360-pH5.7	45.56 ± 0.33 ^b^	26.46 ± 0.26 ^a^	20.95 ± 0.03 ^a^	172.4 ± 0.1 ^a^	138.5 ± 0.8 ^a^	9.8 ± 0.1 ^a^	587.8 ± 7.3 ^k^

^a–m^ Mean values in a column not sharing a common lowercase superscript letter are significantly different (*p* < 0.05).

**Table 2:** Amount of water-soluble extract (WSE; in g, from a mixture of 15 g cheese and 30 g water), protein content (%, *w*/*w*) of the WSE and protein content (%, *w*/*w*) of ultracentrifugal (100,000× *g* for 60 min at 20 °C) supernatant of the WSE of Gouda cheese and of processed cheese (PC) prepared therefrom with 180 or 360 mEq/kg of the calcium sequestering salts (CSS) disodium phosphate (DSP), disodium pyrophosphate (DSPP), sodium hexametaphosphate (SHMP) or trisodium citrate (TSC) and adjusted to pH 5.2 or 5.7. Values are mean ± standard deviation (*n* = 3).

Sample	Amount of WSE (g)	Protein in WSE (%)	Protein in Supernatant (%)
Gouda cheese	23.05 ± 1.30 ^de^	1.42 ± 0.04 ^b^	1.29 ± 0.08 ^ab^
PC-DSP-180-pH5.2	23.30 ± 0.29 ^e^	1.51 ± 0.01 ^bc^	1.37 ± 0.02 ^ab^
PC-DSPP-180-pH5.2	23.40 ± 0.62 ^e^	1.15 ± 0.02 ^a^	1.14 ± 0.02 ^a^
PC-SHMP-180-pH5.2	14.59 ± 0.53 ^b^	3.01 ± 0.08 ^g^	2.52 ± 0.39 ^f^
PC-TSC-180-pH5.2	19.01 ± 0.47 ^c^	1.50 ± 0.03 ^bc^	1.40 ± 0.04 ^ab^
PC-DSP-360-pH5.2	19.69 ± 1.30 ^c^	1.68 ± 0.04 ^cd^	1.59 ± 0.04 ^bc^
PC-DSPP-360-pH5.2	14.61 ± 1.15 ^b^	1.89 ± 0.07 ^d^	1.81 ± 0.04 ^cd^
PC-SHMP-360-pH5.2	13.78 ± 0.14 ^b^	5.57 ± 0.16 ^j^	5.46 ± 0.14 ^ij^
PC-TSC-360-pH5.2	15.32 ± 1.01 ^b^	3.11 ± 0.15 ^g^	2.99 ± 0.13 ^g^
PC-DSP-180-pH5.7	20.55 ± 0.60 ^cd^	2.13 ± 0.01 ^e^	2.11 ± 0.01 ^de^
PC-DSPP-180-pH5.7	15.46 ± 1.26 ^b^	2.49 ± 0.08 ^f^	2.39 ± 0.01 ^ef^
PC-SHMP-180-pH5.7	6.37 ± 0.27 ^a^	5.15 ± 0.01 ^i^	5.14 ± 0.01 ^i^
PC-TSC-180-pH5.7	8.25 ± 0.17 ^a^	5.14 ± 0.02 ^i^	5.10 ± 0.00 ^i^
PC-DSP-360-pH5.7	16.09 ± 1.09 ^b^	4.74 ± 0.03 ^h^	4.72 ± 0.03 ^h^
PC-DSPP-360-pH5.7	24.94 ± 1.13 ^ef^	6.43 ± 0.07 ^l^	6.36 ± 0.11 ^l^
PC-SHMP-360-pH5.7	26.12 ± 1.12 ^f^	6.49 ± 0.04 ^l^	6.08 ± 0.01 ^kl^
PC-TSC-360-pH5.7	28.92 ± 0.17 ^g^	6.12 ± 0.16 ^k^	5.79 ± 0.26 ^jk^

^a–l^ Mean values in a column not sharing a common lowercase superscript letter are significantly different (*p* < 0.05).

**Table 3:** Concentration of Ca, P, Mg and Na (all in mM) in the water-soluble extract (WSE), in the ultracentrifugal (100,000× *g* for 60 min at 20 °C) supernatant of the WSE and 10 kDa permeate of the ultracentrifugal supernatant of Gouda cheese and PC samples prepared therefrom with a pH of 5.2 and 5.7 and with 180 or 360 mEq/kg of the calcium sequestering salts (CSS) disodium phosphate (DSP), disodium pyrophosphate (DSPP), sodium hexametaphosphate (SHMP) or trisodium citrate (TSC). Values are mean ± standard deviation (*n* = 3).

Sample	Ca (mM)			P (mM)			Na (mM)			Mg (mM)		
	WSE	Supernatant	10 kDa	WSE	Supernatant	10 kDa	WSE	Supernatant	10 kDa	WSE	Supernatant	10 kDa
Gouda cheese	34.43 ± 0.2 ^kB^	34.2 ± 0.2 ^kB^	31.9 ± 0.2 ^kA^	13.1 ± 0.1 ^aC^	12.2 ± 0.1 ^aB^	8.3 ± 0.1 ^aA^	106.4 ± 2.2 ^aB^	101.0 ± 0.3 ^aAB^	95.2 ± 1.1 ^aA^	3.8 ± 0.1 ^mB^	3.7 ± 0.1 ^mB^	3.5 ± 0.1 ^iA^
PC-DSP-180-pH5.2	20.6 ± 0.3 ^hB^	20.4 ± 0.1 ^hB^	19.1 ± 0.2 ^jA^	23.4 ± 0.3 ^cdB^	23.1 ± 0.1 ^cdB^	19.2 ± 0.3 ^dA^	136.9 ± 1.8 ^bA^	133.4 ± 0.9 ^bA^	132.6 ± 1.1 ^bA^	3.4 ± 0.1 ^jkB^	3.3 ± 0.1 ^ijB^	3.1 ± 0.1 ^hA^
PC-DSPP-180-pH5.2	15.1 ± 0.1 ^eC^	14.4 ± 0.1 ^fB^	13.6 ± 0.1 ^fgA^	22.4 ± 0.1 ^bcC^	22.0 ± 0.1 ^cB^	18.7 ± 0.1 ^dA^	156.5 ± 4.2 ^dB^	140.3 ± 2.6 ^cA^	136.0 ± 2.6 ^bA^	1.8 ± 0.1 ^dB^	1.7 ± 0.0 ^cA^	1.7 ± 0.0 ^dA^
PC-SHMP-180-pH5.2	17.4 ± 0.5 ^fgB^	16.8 ± 0.5 ^gB^	13.8 ± 0.3 ^gA^	42.5 ± 2.1 ^gB^	41.8 ± 1.6 ^hB^	32.2 ± 0.9 ^iA^	179.3 ± 1.8 ^fB^	175.4 ± 0.7 ^fgAB^	170.2 ± 1.0 ^eA^	2.2 ± 0.1 ^fB^	2.2 ± 0.1 ^eB^	1.9 ± 0.0 ^eA^
PC-TSC-180-pH5.2	21.5 ± 0.6 ^hA^	20.9 ± 0.2 ^hA^	19.7 ± 0.4 ^jA^	26.7 ± 0.6 ^dB^	26.3 ± 0.4 ^eB^	21.3 ± 0.1 ^eA^	148.4 ± 0.3 ^cB^	147.4 ± 0.2 ^dB^	143.6 ± 0.6 ^cA^	3.6 ± 0.1 ^lA^	3.5 ± 0.1 ^klA^	3.4 ± 0.1 ^ijA^
PC-DSP-360-pH5.2	11.8 ± 0.1 ^dB^	11.7 ± 0.1 ^dB^	10.8 ± 0.1 ^dA^	35.9 ± 0.2 ^efB^	35.5 ± 0.1 ^gB^	31.3 ± 0.4 ^iA^	202.9 ± 2.1 ^hiB^	200.0 ± 0.1 ^hiAB^	194.9 ± 1.0 ^hiA^	3.3 ± 0.1 ^ijkB^	3.2 ± 0.1 ^hiB^	3.0 ± 0.1 ^hA^
PC-DSPP-360-pH5.2	6.2 ± 0.2 ^aA^	5.9 ± 0.2 ^aA^	5.6 ± 0.3 ^bA^	37.4 ± 0.3 ^fB^	36.7 ± 0.6 ^gB^	31.8 ± 0.3 ^iA^	188.2 ± 4.1 ^gB^	169.9 ± 2.3 ^fA^	167.5 ± 1.7 ^eA^	0.5 ± 0.0 ^aA^	0.5 ± 0.0 ^aA^	0.5 ± 0.1 ^bA^
PC-SHMP-360-pH5.2	29.3 ± 1.2 ^jB^	28.4 ± 1.5 ^jB^	16.5 ± 0.4 ^iA^	120.4 ± 2.6 ^iB^	119.0 ± 1.3 ^jB^	83.7 ± 0.9 ^kA^	201.8 ± 2.3 ^hiB^	196.8 ± 2.7 ^hiAB^	190.9 ± 1.4 ^ghA^	2.5 ± 0.1 ^gB^	2.4 ± 0.1 ^fB^	1.9 ± 0.1 ^eA^
PC-TSC-360-pH5.2	14.3 ± 0.2 ^eB^	14.1 ± 0.3 ^efB^	13.2 ± 0.1 ^fgA^	37.7 ± 0.1 ^fB^	37.3 ± 0.1 ^gB^	31.1 ± 0.6 ^iA^	216.4 ± 2.2 ^kA^	214.3 ± 3.4 ^kA^	209.3 ± 1.9 ^jA^	3.5 ± 0.2 ^klA^	3.4 ± 0.1 ^jkA^	3.3 ± 0.1 ^iA^
PC-DSP-180-pH5.7	13.8 ± 0.1 ^eB^	13.5 ± 0.1 ^efB^	11.2 ± 0.2 ^dA^	19.1 ± 0.1 ^bB^	18.9 ± 0.2 ^bB^	13.0 ± 0.1 ^bA^	166.9 ± 1.7 ^eB^	160.9 ± 3.6 ^eAB^	153.5 ± 1.4 ^dA^	3.1 ± 0.1 ^hB^	3.0 ± 0.2 ^gB^	2.5 ± 0.1 ^gA^
PC-DSPP-180-pH5.7	10.4 ± 0.4 ^cA^	10.3 ± 0.4 ^cA^	8.9 ± 0.4 ^cA^	21.9 ± 0.9 ^bcB^	21.9 ± 0.9 ^cB^	15.7 ± 0.3 ^cA^	185.8 ± 2.4 ^fgB^	177.1 ± 2.3 ^gAB^	172.1 ± 2.5 ^eA^	1.6 ± 0.1 ^cA^	1.6 ± 0.1 ^bA^	1.4 ± 0.1 ^cA^
PC-SHMP-180-pH5.7	14.1 ± 0.1 ^eC^	12.9 ± 0.1 ^deB^	9.0 ± 0.1 ^cA^	41.7 ± 0.2 ^gC^	41.1 ± 0.1 ^hB^	27.1 ± 0.1 ^gA^	162.2 ± 0.3 ^deC^	157.6 ± 0.2 ^eB^	146.9 ± 0.1 ^cA^	2.0 ± 0.1 ^eC^	1.9 ± 0.1 ^dB^	1.5 ± 0.1 ^cA^
PC-TSC-180-pH5.7	16.5 ± 0.1 ^fB^	16.3 ± 0.1 ^gB^	12.2 ± 0.2 ^eA^	24.66 ± 0.1 ^cdB^	24.5 ± 0.2 ^deB^	12.4 ± 0.1 ^bA^	164.4 ± 0.2 ^eB^	163.4 ± 0.3 ^eB^	141.9 ± 0.3 ^cA^	3.3 ± 0.1 ^ijB^	3.3 ± 0.1 ^ijB^	2.6 ± 0.1 ^gA^
PC-DSP-360-pH5.7	8.0 ± 0.1 ^bB^	7.6 ± 0.2 ^bB^	5.5 ± 0.1 ^bA^	36.7 ± 0.1 ^fB^	35.8 ± 0.4 ^gB^	24.4 ± 0.5 ^fA^	196.6 ± 2.4 ^hB^	194.2 ± 2.4 ^hB^	182.4 ± 1.9 ^fA^	3.2 ± 0.1 ^hiB^	3.1 ± 0.1 ^ghB^	2.4 ± 0.1 ^fA^
PC-DSPP-360-pH5.7	25.5 ± 1.0 ^iB^	4.7 ± 0.1 ^aA^	3.0 ± 0.1 ^aA^	72.1 ± 2.7 ^hC^	42.3 ± 0.2 ^hB^	28.8 ± 0.1 ^hA^	208.8 ± 2.3 ^ijB^	205.3 ± 0.3 ^jB^	197.2 ± 1.3 ^iA^	1.5 ± 0.1 ^bC^	0.4 ± 0.0 ^aB^	0.3 ± 0.0 ^aA^
PC-SHMP-360-pH5.7	35.5 ± 0.2 ^kC^	23.7 ± 0.1 ^iB^	12.9 ± 0.2 ^fA^	132.6 ± 0.2 ^jC^	116.3 ± 0.6 ^iB^	77.3 ± 0.5 ^jA^	201.8 ± 2.9 ^hiB^	195.3 ± 4.4 ^hiAB^	187.0 ± 1.9 ^fgA^	2.6 ± 0.1 ^gC^	2.2 ± 0.1 ^eB^	1.6 ± 0.1 ^dA^
PC-TSC-360-pH5.7	18.5 ± 0.1 ^gC^	17.3 ± 0.2 ^gB^	15.7 ± 0.3 ^hA^	33.1 ± 0.9 ^eB^	31.9 ± 1.1 ^fB^	18.2 ± 0.8 ^dA^	212.6 ± 2.6 ^jkBA^	202.1 ± 1.8 ^ijAB^	193.8 ± 3.9 ^hiA^	3.2 ± 0.1 ^hiA^	3.1 ± 0.1 ^ghA^	3.0 ± 0.2 ^hA^

^a–m^ Mean values in a column not sharing a common lowercase superscript letter are significantly different (*p* < 0.05). ^A–C^ Mean values in a row not sharing a common uppercase superscript letter are significantly different (*p* < 0.05)

**Table 4:** Percentage of Ca, P and Mg in water-soluble extract (WSE) (expressed as a % of total Ca, P and Mg in cheese) and 10 kDa permeate (% of salts in WSE) of Gouda cheese and different processed cheese samples having pH 5.2 and 5.7 and prepared with 180 or 360 mEq/kg of the calcium sequestering salts (CSS) disodium phosphate (DSP), disodium pyrophosphate (DSPP), sodium hexametaphosphate (SHMP) or trisodium citrate (TSC). Values are mean ± standard deviation (*n* = 3).

Samples	Ca		P		Mg	
	in WSE (% of Total)	in 10 kDa Permeate (% of Ca in WSE)	in WSE (% of Total)	in 10 kDa Permeate (% of P in WSE)	in WSE (% of Total)	in 10 kDa Permeate (% of Mg in WSE)
Gouda cheese	46.8 ± 0.5 ^l^	93.5 ± 0.2 ^k^	22.1 ± 0.3 ^a^	68.7 ± 0.3 ^e^	86.5 ± 0.2 ^jk^	93.9 ± 0.4 ^g^
PC-DSP-180-pH5.2	30.7 ± 0.4 ^i^	93.6 ± 0.4 ^k^	30.4 ± 0.2 ^c^	83.5 ± 0.3 ^i^	84.6 ± 0.1 ^ij^	93.5 ± 0.2 ^g^
PC-DSPP-180-pH5.2	22.5 ± 0.1 ^f^	94.1 ± 0.2 ^l^	25.9 ± 0.4 ^b^	84.8 ± 0.2 ^i^	46.1 ± 0.3 ^c^	95.1 ± 0.3 ^hi^
PC-SHMP-180-pH5.2	26.5 ± 0.6 ^g^	82.1 ± 0.3 ^g^	35.3 ± 0.5 ^e^	77.0 ± 0.2 ^g^	58.2 ± 0.5 ^e^	86.1 ± 0.2 ^e^
PC-TSC-180-pH5.2	32.9 ± 0.8 ^j^	94.3 ± 0.7 ^l^	48.9 ± 0.6 ^g^	81.2 ± 0.5 ^h^	92.2 ± 0.8 ^l^	95.6 ± 0.4 ^i^
PC-DSP-360-pH5.2	17.9 ± 0.2 ^d^	92.3 ± 0.4 ^j^	36.5 ± 0.3 ^e^	88.2 ± 0.3 ^k^	82.8 ± 0.3 ^ij^	92.9 ± 0.2 ^g^
PC-DSPP-360-pH5.2	9.5 ± 0.4 ^a^	94.3 ± 0.3 ^l^	31.9 ± 0.2 ^cd^	86.5 ± 0.2 ^j^	13.7 ± 0.4 ^a^	94.0 ± 0.3 ^g^
PC-SHMP-360-pH5.2	45.5 ± 1.3 ^l^	58.3 ± 1.0 ^b^	64.1 ± 0.9 ^i^	70.3 ± 0.7 ^f^	64.4 ± 1.1 ^f^	76.9 ± 0.8 ^b^
PC-TSC-360-pH5.2	22.1 ± 0.1 ^ef^	93.4 ± 0.6 ^k^	72.6 ± 0.3 ^j^	83.6 ± 0.4 ^i^	90.5 ± 0.3 ^kl^	95.5 ± 0.5 ^i^
PC-DSP-180-pH5.7	20.5 ± 0.1 ^e^	82.5 ± 0.3 ^g^	25.2 ± 0.2 ^b^	68.7 ± 0.3 ^e^	78.1 ± 0.4 ^h^	83.7 ± 0.2 ^d^
PC-DSPP-180-pH5.7	15.4 ± 0.7 ^c^	85.6 ± 0.6 ^h^	24.9 ± 0.7 ^b^	71.3 ± 0.4 ^f^	39.9 ± 0.5 ^b^	89.9 ± 0.3 ^f^
PC-SHMP-180-pH5.7	21.2 ± 0.1 ^ef^	70.1 ± 0.3 ^d^	34.3 ± 0.2 ^de^	66.0 ± 0.1 ^c^	52.1 ± 0.2 ^d^	76.2 ± 0.2 ^b^
PC-TSC-180-pH5.7	24.8 ± 0.1 ^g^	74.7 ± 0.3 ^f^	45.0 ± 0.4 ^f^	50.6 ± 0.1 ^a^	86.5 ± 0.5 ^jk^	78.3 ± 0.3 ^c^
PC-DSP-360-pH5.7	11.9 ± 0.1 ^b^	72.8 ± 0.3 ^e^	37.1 ± 0.3 ^e^	68.1 ± 0.2 ^e^	82.1 ± 0.2 ^i^	76.2 ± 0.1 ^b^
PC-DSPP-360-pH5.7	39.1 ± 1.4 ^k^	64.4 ± 1.1 ^c^	60.1 ± 1.3 ^h^	68.0 ± 1.0 ^e^	37.3 ± 0.9 ^b^	66.8 ± 0.9 ^a^
PC-SHMP-360-pH5.7	54.7 ± 0.3 ^m^	54.5 ± 0.5 ^a^	73.5 ± 0.6 ^j^	66.5 ± 0.4 ^d^	72.5 ± 0.4 ^g^	75.1 ± 0.4 ^b^
PC-TSC-360-pH5.7	28.3 ± 0.1 ^h^	90.5 ± 0.2 ^i^	63.3 ± 0.3 ^i^	57.1 ± 0.4 ^b^	86.1 ± 0.5 ^ij^	94.5 ± 0.3 ^gh^

^a–m^ Mean values in a column not sharing a common lowercase superscript letter are significantly different (*p* < 0.05).

**Table 5:** Formulations for processed cheese samples prepared with Gouda cheese, water, lactic acid or NaOH and 180 or 360 mEq/kg of the calcium sequestering salts (CSS) disodium phosphate (DSP), disodium pyrophosphate (DSPP), sodium hexametaphosphate (SHMP) or trisodium citrate (TSC).

Samples	Gouda Cheese (g)	CSS (g)	Water (g)	Lactic Acid (g)	NaOH (g)	Total Weight (g)
PC-DSP-180-pH5.2	262.6	2.6	33.5	1.3	-	300.0
PC-DSPP-180-pH5.2	261.8	3.0	34.2	-	1.0	300.0
PC-SHMP-180-pH5.2	257.5	5.5	36.8	0.2	-	300.0
PC-TSC-180-pH5.2	258.9	4.6	34.2	2.3	-	300.0
PC-DSP-360-pH5.2	258.2	5.2	34.1	2.5	-	300.0
PC-DSPP-360-pH5.2	256.7	6.0	34.8	-	2.5	300.0
PC-SHMP-360-pH5.2	248.1	11.0	40.4	0.5	-	300.0
PC-TSC-360-pH5.2	251.1	9.2	36.5	3.2	-	300.0
PC-DSP-180-pH5.7	262.6	2.6	34.3	-	0.5	300.0
PC-DSPP-180-pH5.7	261.8	3.0	32.0	-	3.2	300.0
PC-SHMP-180-pH5.7	257.5	5.5	35.3	-	1.7	300.0
PC-TSC-180-pH5.7	258.9	4.6	36.3	0.2	-	300.0
PC-DSP-360-pH5.7	258.2	5.2	35.9	0.7	-	300.0
PC-DSPP-360-pH5.7	256.7	6.0	32.5	-	4.8	300.0
PC-SHMP-360-pH5.7	248.1	11.0	38.9	-	2.0	300.0
PC-TSC-360-pH5.7	251.1	9.2	38.5	1.2	-	300.0

## Data Availability

Data are contained within the article.
